# Preparation, Characterization, and In Vitro Evaluation of Resveratrol-Loaded Cellulose Aerogel

**DOI:** 10.3390/ma13071624

**Published:** 2020-04-01

**Authors:** Lili Qin, Xinyu Zhao, Yiwei He, Hongqiang Wang, Hanjing Wei, Qiong Zhu, Ting Zhang, Yao Qin, Ai Du

**Affiliations:** 1 Sports and Health Research Center, Department of Physical Education, Tongji University, Shanghai 200092, China; 1831833@tongji.edu.cn (X.Z.);; 2 School of Physics Science and Engineering, Tongji University, Shanghai 200092, China; 3 School of Life Sciences and Technology, Tongji University, Shanghai 200092, China

**Keywords:** TEMPO-oxidized cellulose, aerogel, resveratrol, drug carrier

## Abstract

Resveratrol is a natural active ingredient found in plants, which is a polyphenolic compound and has a variety of pharmaceutical uses. Resveratrol-loaded TEMPO-oxidized cellulose aerogel (RLTA) was prepared using a freeze-drying method, employing high speed homogenization followed by rapid freezing with liquid nitrogen. RLTAs were designed at varying drug–cellulose aerogel ratios (1:2, 2:3, 3:2, and 2:1). It could be seen via scanning electron microscopy (SEM) that Res integrated into TEMPO-oxidized cellulose (TC) at different ratios, which changed its aggregation state and turned it into a short rod-like structure. Fourier transform infrared (FTIR) spectra confirmed that the RLTAs had the characteristic peaks of TC and Res. In addition, X-ray diffraction (XRD) demonstrated that the grain size of RLTA was obviously smaller than that of pure Res. RLTAs also had excellent stability in both simulated gastric fluid and phosphate buffer solution. The drug release rate was initially completed within 5 h under a loading rate of 30.7 wt%. The results of an MTT assay showed the low toxicity and good biocompatibility of the RLTAs. TC aerogel could be a promising drug carrier that may be widely used in designing and preparing novel biomedicine.

## 1. Introduction

Aerogel is a kind of material with extremely low density, high porosity, high specific surface area, and three-dimensional open networks [1,2,3,4]. Kistler first presented the composition of aerogels including oxides of silicon, aluminum, tin, thorium, iron, tungsten and organic compounds such as cellulose, cellulose nitrate, and gelatin [5,6]. For a long time, as a representative of inorganic aerogels, silica aerogels have been widely studied and applied because of their low cost and good structure and properties [7,8]. In the field of biomedicine, previous studies have shown the diffusion mechanism of drugs in silica aerogels [9,10], and that silica nanoparticle-loaded drugs have had good targeted therapeutic effects on diseases [11,12]. In recent years, considering the degradability of materials, scientists have turned their attention to the development of natural polymers [13].

Cellulose aerogel has, not only the typical characteristics of aerogels, but also outstanding biocompatibility, which has been widely studied and applied as reaction templates, adsorption materials, and carbon aerogel precursors [7]. For example, cellulose aerogel has been used as a catalyst or a catalyst matrix, which can provide a stable network structure, and particles were dispersed uniformly without aggregation [14]. Cellulose aerogels can also be used as an amphiphilic superabsorbent, which is a good choice for wastewater clarification [15,16]. Recently, the application potential of cellulose has also been gradually studied in the field of biomedicine. Most studies have been on wound dressing [17], or tissue engineering scaffolds [18,19,20]. However, cellulose aerogel was also a promising carrier material for drugs, enzymes, and proteins [21]. It can play a role in controlling release [22] and targeted transportation [10]. In addition, the stability of the drug can also be improved [23].

Resveratrol (Res) has a variety of pharmaceutical activities, such as anti-inflammatory, antioxidant, anti-aging, anti-tumor, and cardiovascular protection [24]. However, its pharmacokinetic properties are less favorable, since the use of pure resveratrol can easily cause aggregation. Moreover, Res is difficult to disperse in water, which can lead to the solubility problems for oral administration. In order to overcome these problems, looking for a suitable carrier has attracted many research interests in recent years [25,26].

In this study, compared with previous studies on drug-loaded system release [27,28], we found that the grain size of the system was significantly smaller than that of pure drugs, which was beneficial for the drug absorption in the body. In addition, unlike previous low-temperature cellulose preparation conditions [29], the preparation process was simplified by using TEMPO-oxidized cellulose (TC) as a raw material. We mixed TC with Res in different mass ratios, then quickly froze the mixtures in liquid nitrogen, and finally freeze-dried to obtain the resveratrol-loaded TEMPO-oxidized cellulose aerogel (RLTA). Without changing the properties of TC and Res, Res was well dispersed and the grain size was greatly reduced. In vitro experiments were conducted to study the release behavior of artificial gastric juice and phosphate buffer solution in different proportions. It was observed that RLTA could be stably presented and released without being affected by pH (pH = 1.5 and 7.4). Furthermore, the cytotoxicity effect of RLTAs was evaluated on TC-28a2 human cartilage cells, indicating a good performance for osteoarthritis treatment. It was expected that cellulose aerogel as a high-performance drug carrier could be widely used in the pharmaceutical field.

## 2. Materials and Methods

### 2.1. Material

Trans-resveratrol (≥99%, Res) was purchased from Aladdin Reagent (Shanghai, China) Co., Ltd. Ethanol (≥99.7%, EtOH) was purchased from Sinopharm Chemical Reagent Co., Ltd., Shanghai, China. Deionized water was obtained from the School of Environment and Engineering, Tongji University. Phosphate buffered saline and simulated gastric fluid (SGF) were purchased from Scientific Phygene (Fuzhou, China). TEMPO-oxidized cellulose (TC) was purchased from Ningbo Enerol Nanotechnology Ltd. (Ningbo, China).

### 2.2. Preparations of TC Aerogels Loaded with Res

TC (45 mg) was dissolved in deionized water (5.625 mL) to form a homogeneous solution, A. Res (90 mg) was diluted with alcohol (2 mL) to form solution B. Solutions A and B were mixed with mass ratios of 1:0, 1:2, 2:3, 3:2, 2:1, and 0:1. After further stirring, the mixtures were quickly frozen with liquid nitrogen and then freeze-dried.

### 2.3. Freeze-Drying of RLTAs and Res

In order to remove the solvents water and alcohol, the frozen samples needed to be dried. The first drying was performed at a temperature of −60 °C and a pressure of less than 100 Pa for 24 h. Then a second drying process was performed for 24 h, and the pressure dropped to 9.8 Pa when samples were taken out. Finally, a loose and dry milky white solid was obtained.

The Res was dissolved in alcohol and freeze-dried for characterizations.

### 2.4. Characterizations

The samples were sprayed with gold and used for scanning electron microscopy (SEM). The images were obtained by scanning electron microscope (SEM, Philips-XL30FEG, Thermo Fisher Scientific Inc., Hillsboro, OR, USA).

Nitrogen (N_2_) adsorption−desorption isotherms were measured by using ASAP 2020 (Micromertics, Norcross, GA, USA). Before measuring, all samples were degassed at the corresponding temperature under vacuum overnight. The pore size distributions were calculated by the Barrett−Joyner−Halenda (BJH) method. In addition, a pore size distribution test was also performed on the samples using the mercury intrusion method, using AutoPore Iv 9510 (Micromertics, Norcross, GA, USA).

An FTIR spectrometer (Nicolet 6700, Thermo Fisher Scientific Inc., Hillsboro, OR, USA) was used to characterize the functional groups. The sample powders were dispersed into a KBr matrix at a ratio of 1:100 and pressed for characterization. The measured wavenumber range was 4000–900 cm^−1^.

The chemical structures of the samples were analyzed by ^13^C NMR spectroscopy, using a JNM-ECZ600R 600 MHz NMR spectrometer (JEOL, Tokyo, Japan).

The samples were tested by X-ray diffractograms (XRD) using D8 Advance (Bruker, Karlsruhe, Germany). The generator was operated at 20 kV and 50 mA, and the diffracted intensity data were recorded in the 2θ range of 5°–60°.

Res had strong absorption around the wavelength of 306 nm, which was measured by an ultraviolet-visible-infrared (UV-Vis-IR) spectrophotometer (JASCO V-570, JASCO, Kyoto, Japan). The Res calibration curve was drawn by preparing 5 different concentrations of Res solutions, and the equation y = 122.71x + 0.03502 was obtained. The release of RLTAs in artificial gastric juice and phosphate buffer was measured at different times. The actual release was calculated by measuring the absorbance at 306 nm and bringing it into the above equation.

### 2.5. In Vitro Drug Release of RLTAs

The RLTAs with different ratios and Res (35 mg) were placed in 600 mL of phosphate buffer solution and artificial gastric juice for release study, with mechanical stirring at 100 rpm and oil bath heating at 37 °C. Then, 4 mL of suspension were taken and centrifuged at 5 min, 15 min, 30 min, 1 h, 2 h, 3 h, 4 h, 5 h, 6 h, 12 h, and 24 h, respectively. After that, 1 mL of supernatant was diluted and used to calculate the release amount by measuring the absorbance at a wavelength of 306 nm. Then, the remaining suspension was poured into the original solution, with 1 mL of phosphate buffer or artificial gastric fluid added accordingly. In fact, we used the excessive drug (each release sample contained the same amount, 90 mg of Res) and the relatively small amount of solution to simulate a common excess drug dose. Three types of dissolution–diffusion kinetic models were used in an attempt to explain the mechanism of release:

The zero-order model:

M_t_/M_∞_ = kt (1)

The first-order model:

M_t_/M_∞_ = 1 − e^−k1p×t^ (2)

Higuchi model:

Q = [DS(p/λ)(2A − Sp)t]^1/2^ (3)

Zero-order and first-order models are often used to describe dissolution phenomena. The Higuchi model is generally used to explain the controlled release effects of porous materials as a skeleton on drugs. In these equations, M_t_ and M_∞_ are the absolute cumulative amounts of Res released at time t and at infinite time, respectively, k is a constant, and k_1P_ is the dissolution rate constant. Q is the release amount per unit area at time t, D is the diffusion coefficient, p is the porosity in the skeleton, S is the solubility of the drug in the release medium, λ is the bending factor in the skeleton, and A is the drug content in the skeleton per volume.

### 2.6. In Vitro Cytotoxicity of RLTAs

The viability of TC-28a2 chondrocytes in the presence of RLTAs was evaluated by performing MTT (3-(4,5-dimethylthiazol-2-yl)-2,5-diphenyl tetrazolium bromide) assay. The cell lines TC-28a2 were cultured in DMEM/F12, supplemented with 10% FBS, L-glutamine, and antibiotics. They were seeded in 96-well plates at a density of 20,000 cells per well for 24 h in the presence of MTT. Twenty microliters of RLTAs with different concentrations were added into each group of 3 wells, so that the final concentrations in the wells were 2.5, 5, 10, 20, 40 μg/mL and a blank control. After 24 h, 48 h, and 72 h of incubation, 20 μL of MTT solution was added. After 4 h of incubation, we discarded the supernatant and added 150 μL of DMSO to stop the reaction. After 30 min of gently shaking, the absorbance was monitored at 490 nm to calculate the cell survival using a microplate reader. The percentage viability and cytotoxicity were calculated using the following equation:

(4) $$(\mathrm{viability})\;\%=\frac{\mathrm{Absorbance}\;\mathrm{of}\;\mathrm{test}}{\mathrm{Absorbance}\;\mathrm{of}\;\mathrm{control}}\;\times \;100,$$

## 3. Results and Discussion

### 3.1. Synthesis and Morphology of RLTAs

**Figure 1:**
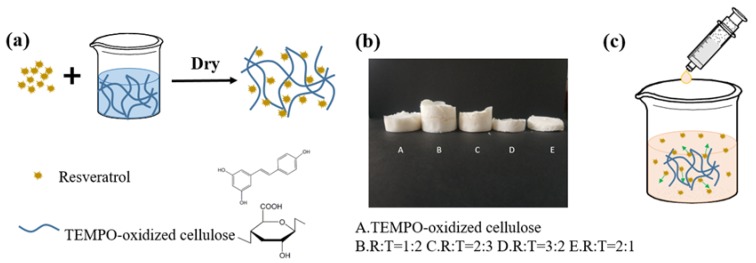
(**a**) Synthesis sketch of Resveratrol-loaded TEMPO-oxidized cellulose aerogel (RLTA), (**b**) digital photographs of RLTA with different Resveratrol (Res) and TEMPO-oxidized cellulose (TC) mass ratios, (**c**) drug release sketch of Res from simulated gastric juice or phosphate buffer.

Figure 1a shows the preparation process of RLTAs. RLTAs were prepared from TC and Res in different ratios. Res was attached to the cellulose skeleton and freeze-dried to obtain a milky white loose structure. As shown in Figure 1b, it can be observed that the pure TC aerogel structure was relatively dense and a yellowish color. As the drug content increased, the structure of RLTA became looser and the color became approximately white, which was closer to the appearance of Res. Figure 1c depicts the release of Res from the TC aerogel in the environment of simulated gastric juice or phosphate buffer, possibly through the diffusion.

### 3.2. The Microstructure of TC Aerogel, Res, and RLTAs

**Figure 2:**
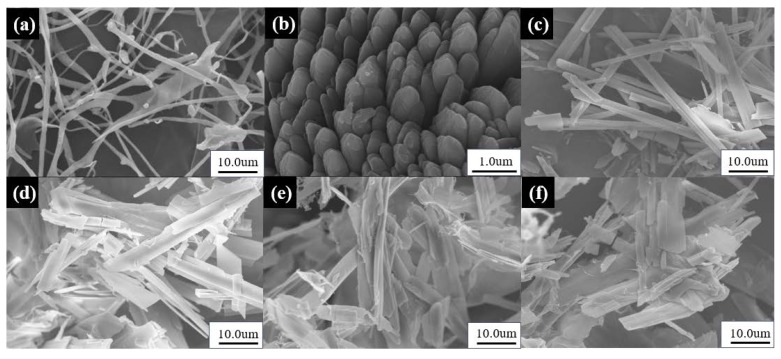
The SEM images of (**a**) TC aerogel, (**b**) Res, (**c**) R:T = 1:2, (**d**) R:T = 2:3, (**e**) R:T = 3:2, (**f**) R:T = 2:1.

As shown in Figure 2, the skeletons of the pure TC aerogel (Figure 2a) was thin, long, and intertwined with multiple fibers [30,31,32,33]. The pure Res had an aggregated rod-like structure (Figure 2b). As the proportion of cellulose increased, the RLTA structure tended to be long, fibrous, and rod-like, which was similar to the pure cellulose. As the proportion of the drug increased, long fiber packs became short and the width of the fiber skeleton increased, forming a sheet-like structure (Figure 2c–f). It was indicated that the TC aerogel’s porous structure was robust enough, and that the Res attached onto the TC surface and filled in the pores in-between the skeletons.

### 3.3. The Pore Size Distribution of TC Aerogel, Res, and RLTA

**Figure 3:**
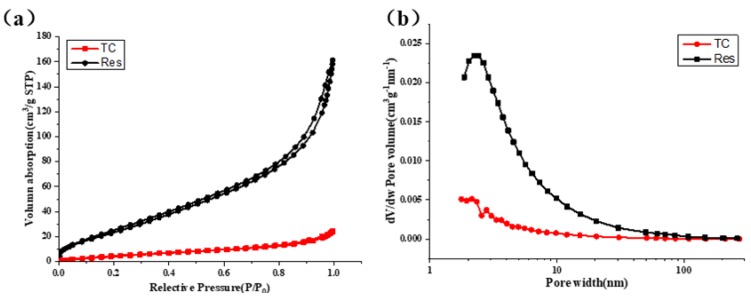
(**a**) Nitrogen adsorption-desorption isotherms of TC and Res. (**b**) The derivative pore size distribution curves for the TC and Res.

**Figure 4:**
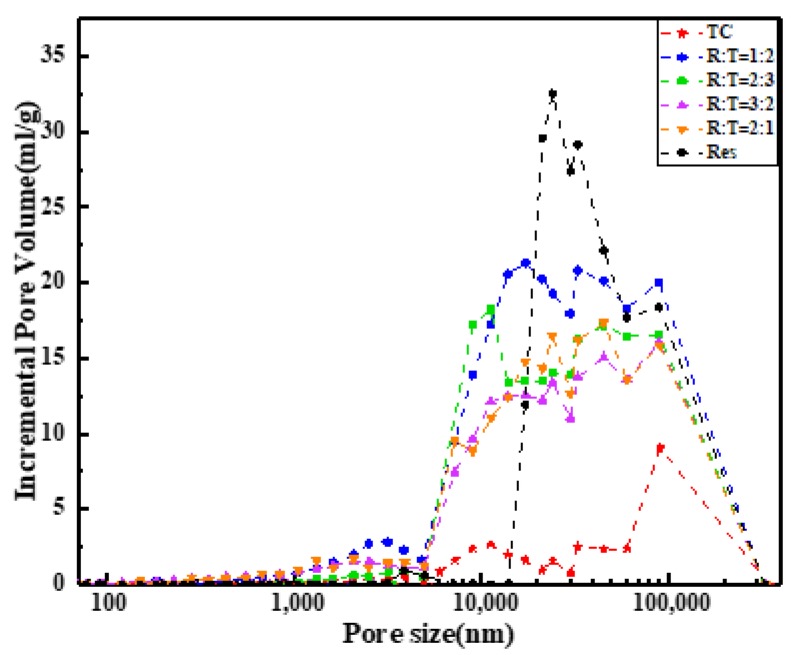
The pore size distribution curve for TC, Res, and the RLTAs.

According to the BET (Brunauer-Emmett-Teller) and BJH (Barret-Johner-Halenda) methods of surface and pore size analysis [34,35], we found that the specific surface area of Res after dissolution-freeze-drying was 95 m^2^/g, and that of the cellulose aerogel was 18.6 m^2^/g. As shown in Figure 3, they had no micropores and only a small amount of mesopores. Therefore, the material was tested for mercury intrusion [36]. We know that the mercury intrusion method relies on external pressure to make mercury overcome the surface tension of the pores of the material to measure its pore size and distribution. As shown in Figure 4, compared with TC, Res had richer macropores. In the RLTA system, a smaller amount of pores appeared richer. We speculated that new pores appeared and the pore diameter became smaller after the action of Res and cellulose.

### 3.4. The FTIR Spectra of the TC Aerogel, Res and RLTAs

**Figure 5:**
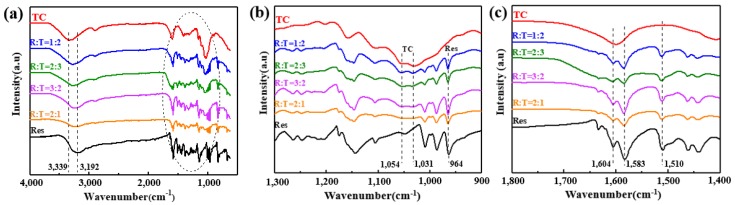
FTIR (**a**) full spectra, (**b**) zoom-in spectra in the band between 1300 to 900 cm^−1^, and (**c**) zoom-in spectra in the band between 1800 to 1400 cm^−1^ of the TC aerogel, Res, and RLTAs.

FTIR full spectra of the TC aerogel, Res and RTLA are shown in Figure 5a. The relatively weak peaks at 1054 cm^−1^ and 1031 cm^−1^ could be attributed to the C–O stretching vibration [37,38,39], and the characteristic peaks related to Res were located at 1604 cm^−1^, 1583 cm^−1^, 1510 cm^−1^, and 964 cm^−1^ [40]. In Figure 5b, RLTAs with different ratios showed the same C–O peaks location as TC aerogels, at 1054 cm^−1^ and 1031 cm^−1^. In Figure 5b,c, the spectra of RLTA showed characteristic peaks of the C=C stretching vibration at 964 cm^−1^, and of the benzene ring vibration at 1604 cm^−1^, 1583 cm^−1^, and 1510 cm^−1^, in accordance with the Res. There were no obvious differences in RLTAs with different ratios. The RLTAs all exhibited the characteristic peaks of TC aerogel and Res, which indicated a successful composite of Res and TC. The band at 3000–4000 cm^−1^ was attributed to the Res–OH stretching vibration with a broad peak around 3192 cm^−1^ that indicated the hydrogen bond between Res molecules. The similar peaks were broadened and shifted to 3242, 3256, 3274, and 3284 cm^−1^ in the spectra of RLTAs with different ratios. We found that broad OH stretching peaks shifted towards the OH bands of pure TC (3339 cm^−1^) with the increasing of TC content. The single peak and significant changes in the OH stretching vibration demonstrated the intermolecular hydrogen bonding between TC and Res hydroxyl groups [41].

### 3.5. The ^13^C NMR Spectra of the TC Aerogel, Res, and RLTAs

**Figure 6:**
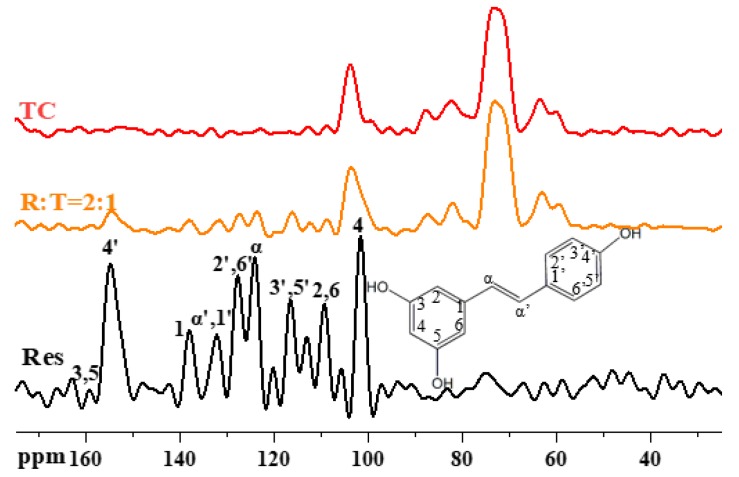
Solid-state CPMAS 13C-NMR spectra of Res, TC, and R:T = 2:1.

We used CPMAS ^13^C-NMR to further explain RLTA interactions. As shown in Figure 6, the Res spectrum was assigned in accordance with the literature [42]. Res peaks were located between 65 and 163 ppm and did not overlap with TC. Res peaks could mainly be found in RLTA except that of Res C-4, which combined with cellulose to form a broad peak at 103.8 ppm. Small changes in chemical shifts of some peaks existed, such as C4′ (from 154.3 ppm in RLTA to 154.8 ppm in Res) and C-2,6 (from 108.7 ppm in RLTA to 109.32 ppm in Res). Considering that the hydroxyl bands of the RLTAs were between those of the TC and Res (3000–4000 cm^−1^ in Figure 5a), the existence of hydrogen bonding interactions between TC and Res could be confirmed.

### 3.6. The XRD Spectra of the TC Aerogel, Res, and RLTAs

**Figure 7:**
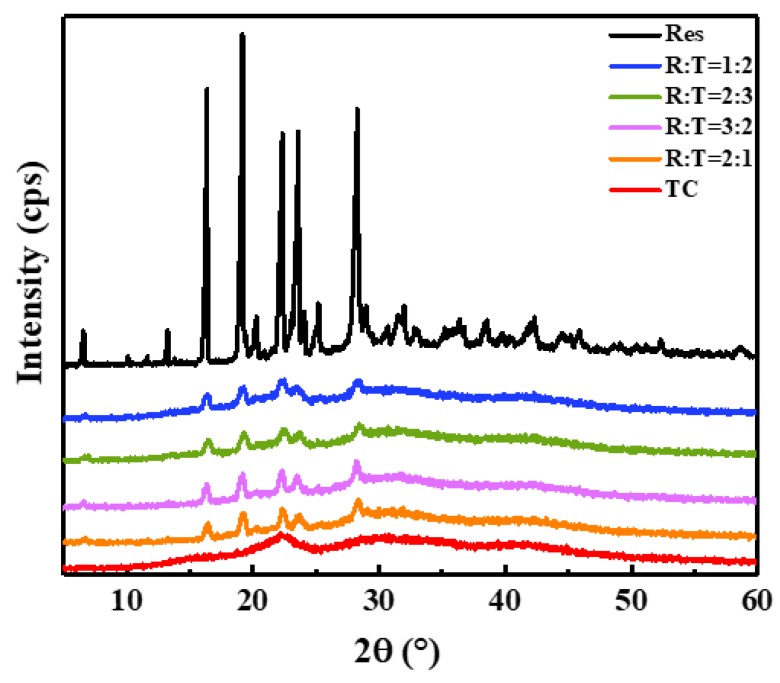
XRD spectra of Res, TC aerogel, and RLTAs.

The X-ray diffraction technique was employed to confirm the XRD patterns of TC, Res, and RLTAs. As shown in Figure 7, TC aerogel could be considered as a disordered nano-crystalline material because the broad halos were similar to the parent cellulose I crystalline peaks [27,43]. When the ratio of TC to Res was different, there were still five obvious Res crystal diffraction peaks of 16.35°, 19.24°, 22.46°, 23.54°, and 28.21° [26,41,44]. It could be seen that the crystal form hardly changed after recombination. The particle size of the sample was analyzed according to the Scherrer formula. The relatively large 2θ angle of 28.5° was selected to calculate the crystalline size for accuracy. The original drug’s crystalline size was 34.3 nm. The size was reduced to 0.3–0.6 nm in different proportions of RLTA. According to the verification experiment of the Scherrer formula, it was concluded that the smaller the crystal plane, the larger the error. Although the calculated value may not have been accurate, the increased FWHM (Full-Width Half-Maximum) still strongly indicated a significant decrease of the crystalline size after recombination.

### 3.7. In Vitro Drug Release of RLTAs

**Figure 8:**
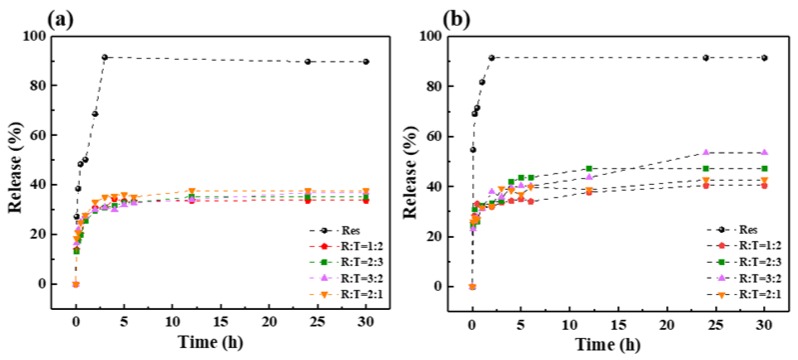
Res and RLTAs in (**a**) simulated gastric juice and (**b**) phosphate buffer.

In order to study the release behavior of TC aerogel loaded with Res, the samples were placed in a simulated gastric fluid (PH = 1.5) and a phosphate buffer solution (PH = 7.4) for observation. As shown in Figure 8, the release amount changed with the increase of the sustained release time. It was found that, whether in the simulated gastric fluid or in the phosphate buffer solution, a burst occurred in the first 15 min, which could be attributed to the desorption of the Res from TC aerogel surface. Subsequently, a sustained and slow release was performed, and initially stop releasing after 5 h. The drug releases were about 35.6% for simulated gastric juice and 49.5% for phosphate buffer, respectively. An equivalent amount of 35 mg Res was used for the release, and it was found that the release amount reached 91% in 5 h. In the simulated gastric juice, the release amount of the samples was not significantly different between different proportions, and the release curves tended to be saturated. It could be seen that there was an increase trend of the release amount with the increase of the drug proportion. In the phosphate buffer solution (PH = 7.4), there was no obvious regularity between different proportions, but the overall release was slightly higher than in the simulated gastric fluid. In short, the RLTAs had no significant difference between different proportions, and could be stably released in artificial gastric juice (PH = 1.5) and phosphate buffer solution (PH = 7.4). The release ratio for RLTAs seemed to be relatively low because of the addition of excessive Res (the content is 90 mg each).

According to results from the literature [45], the saturability of Res in acidic and neutral solutions are 61.8 ug/mL and 59.8 ug/mL, respectively. By calculating the situation (600 mL solution) we used, the dissolved quantity of Res in acidic and neutral solutions should have been about 37.1 mg and 35.9 mg. By considering the different experimental, environmental, and related errors, our results were similar to those values.

### 3.8. Mechanism of Release

Three types of dissolution–diffusion kinetic models (Zero-order, First-order and Higuchi) were used in an attempt to explain the mechanism of release [46,47,48,49]. The correlation coefficients (R) reported in Table 1 were obtained. A higher R value means a better fit to the model. We found that Res conformed to the first-order release model and that the release rate was related to the concentration difference. We also found that during the drug release process in RLTAs, the R values in the first-order and the Higuchi were not very different. Here, we choose to use the Higuchi to analyze based on the release of most porous materials as the skeleton [48]. In this study, the slow dissolution of the drug in the RLTAs system may have been due to the combination of cellulose and the drug. When continuing to dissolve, the solvent needed to pass through the pores. Alternatively, it may have been due to the blocking effect of the cellulose matrix on the drug. It takes time to dissolve the cellulose matrix, which would have continued to release until 5 h.

### 3.9. In Vitro Cytotoxicity of RLTAs

**Figure 9:**
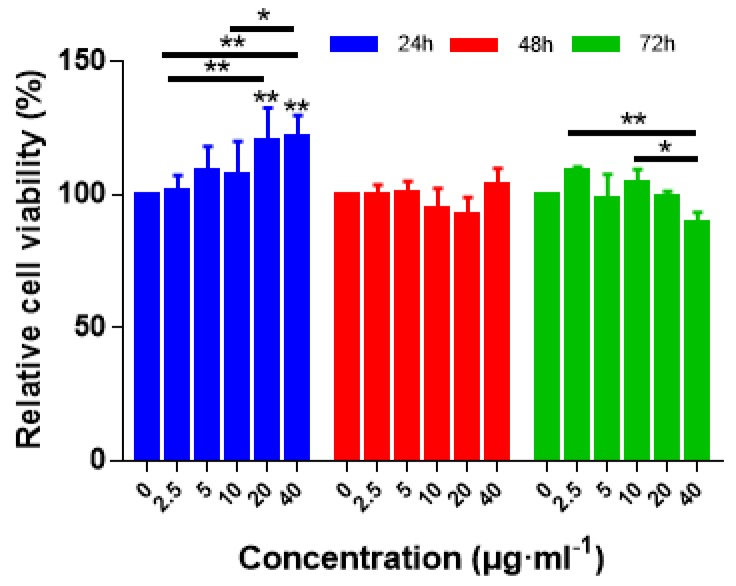
Cell viability of RLTAs (R:T = 2:1) on the growth of the cartilage cells TC-28a2. The cells have been treated with one of three treating times with various concentration for 24, 48, or 72 h at concentrations of 2.5, 5, 10, 20, and 40 μg/mL. Results represent the means of three independent experiments and error bars represent the standard error of the mean. Statistical significance was assessed using two-way ANOVAs with Tukey’s multiple comparison tests (* *p* < 0.05, ** *p* < 0.01).

Moreover, the cytotoxicity of the RLTA were evaluated in cartilage cells TC-28a2 by the standard MTT assay for use in the treatment of osteoarthritis in our subsequent study [50]. Figure 9 shows the effect of RLTA (R:T = 2:1) on the proliferation of cartilage cells at various concentrations. We chose cellulose materials without drugs as the blank control group. It did not show an obvious toxicity, owing to the relative cell viability remaining above 90%, even when the Res concentration was 40 μg/mL. After co-incubating with TC-28a2 cells for 24 h, 48 h, and 72 h, the relative cell viability at each concentration was still higher than 90%. These results indicated that RLTAs were hardly toxic to the TC-28a2 cells and had better biocompatibility for serving as a safe drug-delivery option in the cellular system.

## 4. Conclusions

In this study, it was found that the crystalline size of Res in RLTAs was significantly reduced relative to pure Res. The short rod-like structure of RLTAs could be clearly seen in SEM images. RLTAs had the characteristics of both raw materials, according to the FTIR and ^13^C NMR analysis, indicating the good recombination. XRD results illustrated that the drug loaded on cellulose had significantly small grain size. By simulating the drug release in the gastric fluids or body fluids, it was found that the drug-loaded system showed a good stability and would not be damaged under the gastric acid environment. In addition, RLTAs had good biocompatibility, according to in vitro cytotoxicity results. TC aerogels are expected to be an ideal carrier for various drugs, leading to potential broad applications in the field of biomedicine.

## Figures and Tables

**Table 1:** Correlation coefficients (R) for dynamic release curves.

		R		
Sample	PH Value	Zero-Order	First-Order	Higuchi
Res	7.4	0.836	0.989	0.905
	1.5	0.892	0.971	0.969
R:T = 1:2	7.4	0.779	0.805	0.857
	1.5	0.888	0.963	0.978
R:T = 2:3	7.4	0.789	0.798	0.841
	1.5	0.886	0.985	0.962
R:T = 3:2	7.4	0.845	0.923	0.954
	1.5	0.841	0.997	0.907
R:T = 2:1	7.4	0.792	0.891	0.836
	1.5	0.86	0.939	0.976
